# Efficacy of PD‐1/PD‐L1 inhibitors in patients with advanced non‐small cell lung cancer: A meta‐analysis of randomized clinical trials

**DOI:** 10.1111/1759-7714.13060

**Published:** 2019-04-10

**Authors:** Tzu‐Rong Peng, Ta‐Wei Wu

**Affiliations:** ^1^ Department of Pharmacy, Taipei Tzu Chi Hospital Buddhist Tzu Chi Medical Foundation New Taipei City Taiwan; ^2^ School of Pharmacy, College of Pharmacy Taipei Medical University Taipei City Taiwan

**Keywords:** Non‐small cell lung cancer, programmed death ligand inhibitor, programmed death‐1 receptor inhibitor

## Abstract

**Background:**

This meta‐analysis systematically evaluated the efficacy of PD‐1 and PD‐L1 inhibitors for the treatment of advanced non‐small cell lung cancer (NSCLC) and investigated the efficacy of first‐line therapy and PD‐1 versus PD‐L1 inhibitors.

**Methods:**

PubMed, The Cochrane Library, and Embase were searched up to November 2018 for randomized controlled trials (RCTs) for eligible studies. The outcome of interest was overall survival (OS). The methodology was based on the Preferred Reporting Items for Systematic Reviews and Meta‐Analyses and Cochrane Collaboration guidelines. Data were pooled by using the random effects model and expressed as hazard ratios (HRs) and corresponding 95% confidence intervals (CIs). Heterogeneity was assessed and quantified (*I* ^*2*^).

**Results:**

Seven RCTs were included in this study. PD‐1/PD‐L1 inhibitors achieved superior OS compared to chemotherapy (HR 0.72, 95% CI 0.63–0.82; *P* < 0.0001). OS was superior in previously treated patients compared to untreated patients (HR 0.69, 95% CI 0.63–0.76; HR 0.82, 95% CI 0.47–1.44, respectively). No significant differences in OS were observed between PD‐1 and PD‐L1 inhibitors (HR 0.71, 95% CI 0.59–0.86; HR 0.73, 95% CI 0.63–0.84, respectively).

**Conclusions:**

PD‐1/PD‐L1 inhibitors significantly prolonged the OS of previously treated patients. No significant differences in OS were observed between PD‐1 and PD‐L1 inhibitors.

## Introduction

Immunotherapy is a new therapeutic option for multiple tumor types, including non‐small cell lung cancer (NSCLC). PD‐1 and PD‐L1 inhibitors that target the PD‐1 and PD‐L1 pathways have demonstrated clinical efficacy and safety for the treatment of NSCLC.^1^, ^2^, ^3^, ^4^, ^5^, ^6^, ^7^ The United States Food and Drug Administration (FDA) has approved pembrolizumab for the first‐line treatment of metastatic NSCLC patients with high tumor PD‐L1 expression.^8^, ^9^ Furthermore, according to the results of the CheckMate 026 trial, nivolumab did not result in significantly longer progression‐free survival compared to systemic chemotherapy in patients with previously untreated stage IV or recurrent NSCLC with a PD‐L1 expression level of ≥ 5%. This trial showed that overall survival (OS) in patients treated with nivolumab and chemotherapy was similar.^2^ Atezolizumab and durvalumab are human‐engineered immunoglobulin G1 (IgG1) monoclonal antibodies targeting PD‐L1 and thus have a mechanism of action distinct from anti‐PD‐1 antibodies. They have higher objective response rates (ORRs) in the first‐line treatment of patients with advanced NSCLC. Most studies of these drugs have been phase‐I single‐arm trials.^10^, ^11^, ^12^

Systematic reviews comparing the toxicity profile of PD‐1 versus PD‐L1 inhibitors have shown that overall adverse events (AEs) and grade 3–5 AEs of PD‐1 and PD‐L1 inhibitors are similar.^13^ However, the efficacy of PD‐1 versus PD‐L1 inhibitors remains unknown. Given the inconsistencies in these studies and the unknown parameters, we pooled the hazard ratios (HRs) of OS to determine the efficacy of PD‐1/PD‐L1 inhibitors. We conducted a meta‐analysis to evaluate the OS of patients with advanced NSCLC who were administered PD‐1/PD‐L1 inhibitors. Subgroup analyses of OS were also based on the sequence of treatment (first‐line and second‐line treatment) and PD‐1 versus PD‐L1 inhibitors.

## Methods

### Literature search

We searched online databases, such as PubMed, the Cochrane Library, and Embase, for relevant literature published up to November 2018. The following search terms were used: PD‐1 or PD‐L1 or nivolumab OR pembrolizumab OR atezolizumab OR durvalumab AND non‐small‐cell lung cancer. The search was limited to papers published in English and those related to studies involving humans. Detailed information on the search strategy for eligible studies is provided in the flowchart in the Preferred Reporting Items for Systematic Reviews and Meta‐Analyses guidelines.^14^ Two of the authors independently reviewed the retrieved papers. Any discrepancies between the reviewers were resolved by consensus.

### Data extraction and selection criteria

This study was conducted in accordance with Cochrane Collaboration guidelines.^15^ The following information was extracted: included author/s, year of publication, interventions, number of enrolled patients, doses, and clinical efficacy (HR with 95% confidence intervals [CIs] and *P* values for OS). Trials that met the following criteria were eligible for inclusion: (i) phase II or III randomized controlled trials (RCTs); (ii) trials in which patients were administered an anti‐PD‐1 or anti‐PD‐L‐1 inhibitor for the treatment of advanced NSCLC; and (iii) trials that reported OS as a clinical outcome.

### Quality assessment

Two reviewers independently extracted the baseline and outcome data and assessed the methodological quality of each study by using the risk of bias method recommended by the Cochrane Collaboration.^16^ Several domains were assessed, including the adequacy of the randomization, allocation concealment, blinding of the patients and outcome assessors, length of follow‐up, information provided to the patients regarding study withdrawal, whether intention‐to‐treat analysis was performed, and freedom from other biases.

### Statistical analysis

Statistical analysis was performed according to the Cochrane Handbook for Statistical Review of Interventions, version 5.3.^17^ The meta‐analysis was performed using RevMan software (Cochrane Review Manager Version 5.3, Oxford, UK). Differences between immunotherapy and chemotherapy (docetaxel or platinum‐based chemotherapy) were assessed using a HR with a 95% CI. The random effects model (DerSimonian–Laird method) was used to calculate the pooled HR.^18^ Publication bias was examined using funnel plots. We assessed heterogeneity using a χ^2^ test with *P* < 0.10 considered statistically significant. Heterogeneity was considered low, moderate, or high for *I* ^2^ values of < 25, 25–50, and > 50%, respectively. Results were considered statistically significant with a *P* value of < 0.05.

## Results

### Characteristics of the included trials

**Figure 1:**
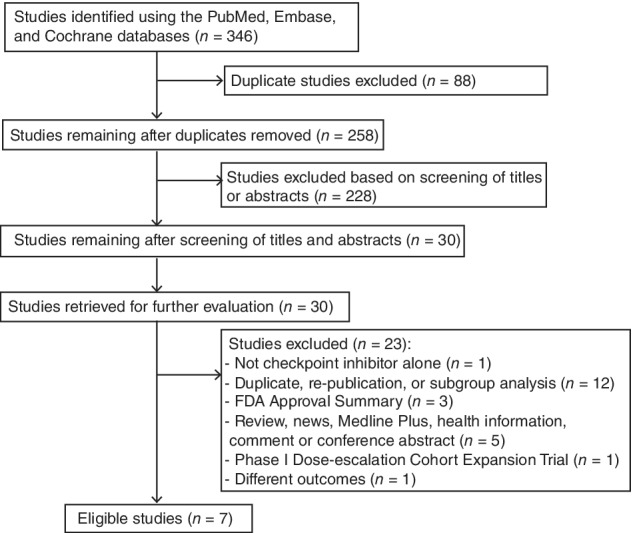
Flowchart describing the inclusion of studies.

A total of 346 publications were identified. After duplicates and screened titles and abstracts were excluded, 30 articles remained for further evaluation. After a full article review was conducted, seven distinct trials were included. The trial selection procedure is shown in Figure 1. A total of seven RCTs were identified, involving 3870 participants with advanced NSCLC. The participants in RCTs were randomized to either receive anti‐PD1/PD‐L1 therapies or chemotherapy. The characteristics of the RCTs are summarized in Table 1. The risk of bias assessment is shown in Table 2.

**Table 1:** Characteristics of studies included in the meta‐analysis

Study	Phase	Design	Line of treatment	Treatment arms (sample size)
Borghaei *et al.* (2015)^3^	III	Open‐label	> 1	Nivolumab 3 mg/kg q2w (*n* = 135) Docetaxel 75 mg/m^2^ q3w (*n* = 137)
Brahmer *et al.* (2015)^4^	III	Open‐label	> 1	Nivolumab 3 mg/kg q2w (*n* = 292) Docetaxel 75 mg/m^2^ q3w (*n* = 290)
Carbone *et al.* (2017)^2^	III	Open‐label	1	Nivolumab 3 mg/kg q2w (*n* = 271) Platinum‐based chemotherapy q3w (*n* = 270)
Herbst *et al.* (2016)^5^	II/III	Open‐label	> 1	Pembrolizumab 2 mg/kg q3w (*n* = 339) Pembrolizumab 10 mg/kg q3w (*n* = 343) Docetaxel 75 mg/m^2^ q3w (*n* = 343)
Reck *et al.* (2016)^1^	III	Open‐label	1	Pembrolizumab (fixed dose of 200 mg q3w) (*n* = 154) Platinum‐based chemotherapy (*n* = 151)
Fehrenbacher *et al.* (2016)^19^	II	Open‐label	> 1	Atezolizumab 1200 mg q3w (*n* = 144) Docetaxel 75 mg/m^2^ q3w (*n* = 143)
Rittmeyer *et al.* (2017)^20^	III	Open‐label	> 1	Atezolizumab 1200 mg q3w (*n* = 425) Docetaxel 75 mg/m^2^ q3w (*n* = 425)

**Table 2:** Quality assessment of 10 randomized controlled trials included

Study	Generation of the allocation sequence	Concealment of the allocation sequence	Blinding of participants and researchers	Blinding of outcome assessment	Incomplete outcome data	Selective reporting	Other bias†
Borghaei *et al.* 3	Low	Low	High	High	Low	Low	Unclear
Brahmer *et al.* 4	Low	Low	High	High	Low	Low	Unclear
Carbone *et al.* 2	Low	Low	High	High	Low	Low	Unclear
Herbst *et al.* 5	Low	Low	High	High	Low	Low	Unclear
Reck *et al.* 1	Low	Low	High	High	Low	Low	Unclear
Fehrenbacher *et al.* 19	Low	Low	High	High	Low	Low	Unclear
Rittmeyer *et al.* 20	Low	Low	High	High	Low	Low	Unclear

† Other bias refers to selective bias and measurement bias.

### Meta‐analysis results of overall survival

**Figure 2:**
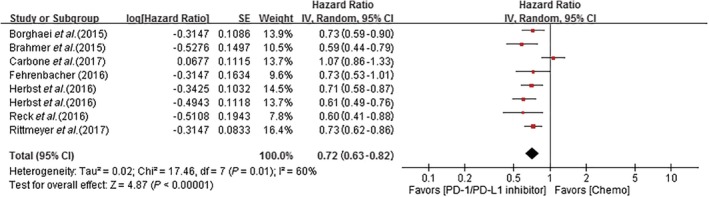
Forest plot of the meta‐analysis of overall survival showing the comparison of PD1/PD‐L1 inhibitors to chemotherapy in non‐small cell lung cancer. The squares represent the hazard ratios of each trial and the horizontal line crossing the squares represents the 95% confidence interval (CI). The diamonds represent the estimated overall effect based on the meta‐analysis random effect of the trials. Chemo, chemotherapy; SE, standard error.

Pooled HRs based on the seven studies revealed a low risk of bias with anti‐PD‐1/PD‐L1 antibody therapies compared to chemotherapy (HR 0.72, 95% CI 0.63–0.82; *P* < 0.0001). The pooled HR for OS using the random effects model is shown in Figure 2.

### Subgroup analysis

Subgroup analysis according to patients who were previously treated or untreated, and administered PD‐1 versus PD‐L1 inhibitors, was performed.

#### Meta‐analysis results of previously treated versus untreated patients

**Figure 3:**
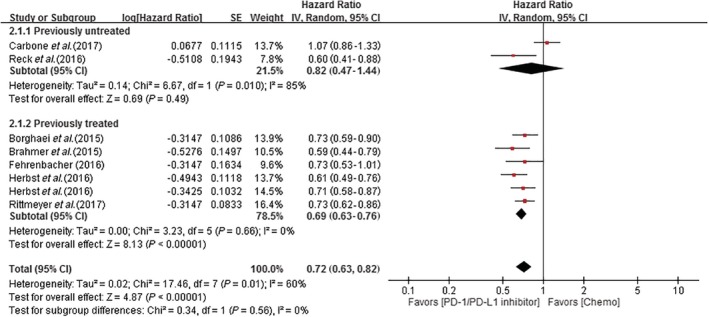
Forest plot of the efficacy of PD‐1/PD‐L1 inhibitors in previously treated versus untreated non‐small cell lung cancer patients. The outcome was hazard ratio (HR) of OS. The squares represent the HRs of each trial and the horizontal line crossing the squares represents the 95% confidence interval (CI). The diamonds represent the estimated overall effect based on the meta‐analysis random effect of the trials. Chemo, chemotherapy; SE, standard error.

A meta‐analysis of all seven trials showed significantly higher OS in previously treated compared to untreated patients (HR 0.69, 95% CI 0.63–0.76; HR: 0.82, 95% CI 0.47–1.44, respectively) (Fig 3).

#### Meta‐analysis results of PD‐1 versus PD‐L1 inhibitors

**Figure 4:**
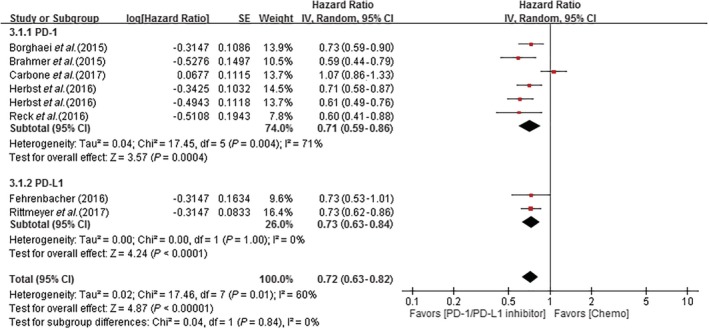
Forest plot of the comparison PD‐1 and PD‐L1 inhibitors. The outcome was HR of OS. The squares represent the HRs of each trial and the horizontal line crossing the squares represents the 95% confidence interval (CI). The diamonds represent the estimated overall effect based on the meta‐analysis random effect of the trials. Chemo, chemotherapy; SE, standard error.

No significant differences were observed in OS between patients treated with PD‐1 or PD‐L1 inhibitors (HR 0.71, 95% CI 0.59–0.86; HR: 0.73, 95% CI 0.63–0.84, respectively) (Fig 4).

### Publication bias

**Figure 5:**
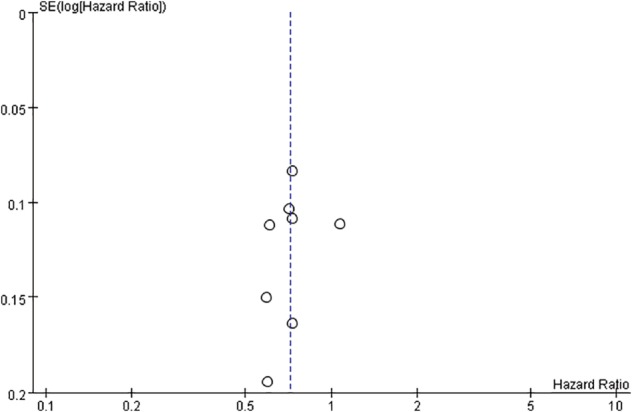
Publication bias funnel plots for overall survival. SE, standard error.

A funnel plot indicated no evidence of substantial publication bias (Fig 5).

## Discussion

Lung cancer is currently a leading cause of cancer death worldwide, with approximately 85% of lung cancer cases attributable to NSCLC.^21^ More than 60% of newly diagnosed patients have locally advanced or metastatic diseases, both conferring a poor prognosis and high mortality.^22^ Patients who have previously received treatment, or develop disease progression or metastatic NSCLC are particularly difficult to treat, and systemic chemotherapy provides only moderate benefits. Immunotherapy drugs, especially PD‐1/PD‐L1 inhibitors, are a new treatment option for NSCLC patients. Several recent studies have examined the therapeutic efficacy using different phases and patient types.

In an analysis of available phase I–III studies, Khunger *et al.* revealed a high ORR and higher rate of immune‐mediated pneumonitis in patients with previously untreated NSCLC compared to patients administered chemotherapy. However, no significant difference was noted in progression‐free survival between previously treated and untreated patients.^23^ In our study, we included the latest phase III RCTs. We found that OS in patients with NSCLC who received first‐line treatment with PD‐1/PD‐L1 inhibitors was not superior to patients administered chemotherapy (HR 0.82, 95% CI 0.47–1.44). Furthermore, the use of PD‐1/PD‐L1 for the treatment of NSCLC in patients who had previously received chemotherapy conferred superior OS than chemotherapy alone (HR 0.69, 95% CI 0.63–0.76).

In a related study, the ORR of PD‐1 and PD‐L1 inhibitors was similar in an unselected population with advanced stage NSCLC. However, the aforementioned studies have some limitations. ORR is a secondary outcome in the current study and the included patients were from clinical studies of different stages. In addition, the systematic review by Pillai *et al.* comparing the toxicity profile of PD‐1 versus PD‐L1 showed that overall and grade 3–5 AEs related to treatment with PD‐1 and PD‐L1 inhibitors were similar (*P* > 0.05).^13^ Our study was undertaken to compare the efficacy of PD‐1 and PD‐L1 inhibitors for the treatment of NSCLC. The results of our meta‐analysis show that PD‐1 and PD‐L1 inhibitors have similar treatment efficacy for NSCLC (HR 0.71, 95% CI 0.59–0.86; HR 0.73, 95% CI 0.63–0.82, respectively; *P* = 0.84, *I* ^2^ = 0%).

The present study had some limitations. First, only two RCTs that investigated the efficacy and safety of anti‐PD‐1/PD‐L1 antibodies for patients with previously untreated advanced NSCLC were available for inclusion, which limited the number of studies available for our meta‐analyses. Second, no phase II or III RCTs have examined atezolizumab and durvalumab as first‐line treatment in patients with advanced NSCLC, which also limited the number of studies available for our meta‐analyses. More RCTs with larger sample sizes are required to confirm these clinical outcomes.

PD‐1/PD‐L1 inhibitors significantly prolong OS in previously treated patients compared to untreated patients. PD‐1/PD‐L1 inhibitors have similar therapeutic effects for the treatment of NSCLC.

## Disclosure

No authors report any conflict of interest.
